# The association of CD81 with tetraspanin-enriched microdomains is not essential for Hepatitis C virus entry

**DOI:** 10.1186/1471-2180-9-111

**Published:** 2009-05-28

**Authors:** Vera Rocha-Perugini, Muriel Lavie, David Delgrange, Jonathan Canton, André Pillez, Julie Potel, Cécile Lecoeur, Eric Rubinstein, Jean Dubuisson, Czeslaw Wychowski, Laurence Cocquerel

**Affiliations:** 1 Institut de Biologie de Lille, CNRS-UMR8161, Université Lille Nord de France, Institut Pasteur de Lille, Lille, France; 2 Institut de Biologie de Lille, CNRS-UMR8090, Université Lille Nord de France, Institut Pasteur de Lille, Lille, France; 3 INSERM-U602, Institut André-Lwoff, Université Paris XI, Hôpital Paul Brousse, Villejuif, France; 4 DKFZ, Tumorvirologie, Virus-Produktion und Entwicklung, Heidelberg, Germany

## Abstract

**Background:**

Three percent of the world's population is chronically infected with hepatitis C virus (HCV) and thus at risk of developing liver cancer. Although precise mechanisms regulating HCV entry into hepatic cells are still unknown, several cell surface proteins have been identified as entry factors for this virus. Among these molecules, the tetraspanin CD81 is essential for HCV entry. Interestingly, CD81 is also required for *Plasmodium *infection. A major characteristic of tetraspanins is their ability to interact with each other and other transmembrane proteins to build tetraspanin-enriched microdomains (TEM).

**Results:**

In our study, we describe a human hepatoma Huh-7 cell clone (Huh-7w7) which has lost CD81 expression and can be infected by HCV when human CD81 (hCD81) or mouse CD81 (mCD81) is ectopically expressed. We took advantage of these permissive cells expressing mCD81 and the previously described MT81/MT81w mAbs to analyze the role of TEM-associated CD81 in HCV infection. Importantly, MT81w antibody, which only recognizes TEM-associated mCD81, did not strongly affect HCV infection. Furthermore, cholesterol depletion, which inhibits HCV infection and reduces total cell surface expression of CD81, did not affect TEM-associated CD81 levels. In addition, sphingomyelinase treatment, which also reduces HCV infection and cell surface expression of total CD81, raised TEM-associated CD81 levels.

**Conclusion:**

In contrast to *Plasmodium *infection, our data show that association of CD81 with TEM is not essential for the early steps of HCV life cycle, indicating that these two pathogens, while using the same molecules, invade their host by different mechanisms.

## Background

Approximately 130 million people are infected worldwide by Hepatitis C Virus (HCV) [1]. Almost 80% of infected patients develop a chronic hepatitis that can in the long term evolve either to liver cirrhosis or hepatocellular carcinoma. Unfortunately, no vaccine is currently available to prevent new infections and the current treatments are not fully efficient [2]. HCV is an enveloped RNA virus mainly targeting liver cells by a mechanism that has yet to be elucidated. For a long time, it has been difficult to study the different steps of the HCV life cycle because of the difficulties in propagating this virus in cell culture. However, a major step in investigating HCV entry was achieved in the development of pseudotyped particles (HCVpp), consisting of native HCV envelope glycoproteins, E1 and E2, assembled onto retroviral core particles [3-5]. More recently, the development of a cell culture system allowing an efficient amplification of HCV (HCVcc) has also been reported [6-8]. This cell culture system allows the study of the whole life cycle of HCV and, together with HCVpp, also permits the characterization of HCV entry mechanisms.

Although the early steps of viral entry have yet to be elucidated, accumulated data suggest several cell surface-expressed molecules as entry factors for HCV (reviewed in [9]). Among these molecules, the tetraspanin CD81 has been shown to play a key role in HCV entry, acting during a post-attachment step [10,11]. Like all members of the tetraspanin family, CD81 is composed of four transmembrane domains, a small extracellular loop (SEL) and a large extracellular loop (LEL), which contains a conserved CCG amino acid motif involved in the formation of disulfide bridges [12]. The CD81 LEL is the critical region for the interaction with the E2 envelope glycoprotein and for virus entry. The role of CD81 in the species restriction of HCV has been extensively studied [13-18], and it has been recently shown that in spite of the absence of *in vitro *interaction between murine CD81 (mCD81) LEL and a soluble form of HCV E2, the ectopic expression of mCD81 in HepG2 cells restored permissivity to HCVpp and, in a lesser extent, to HCVcc [15]. These results suggest that CD81 contributes to, but alone does not define, the species restriction and additional cellular factors are likely involved. Moreover, we have recently shown that EWI-2wint, a new partner of CD81, is able to modulate HCV entry in target cells suggesting that, in addition to the presence of specific entry factors in the hepatocytes, the absence of a specific inhibitor may contribute to the hepatotropism of HCV [19].

Members of the tetraspanin family organize and regroup their associated transmembrane proteins and are involved in various functions such as cell morphology, motility, fusion and signalling [12,20]. A major characteristic of tetraspanins is their ability to interact with each other and with other transmembrane proteins, thus building multi-molecular membrane complexes, collectively referred to as the tetraspanin enriched microdomains (TEM) or tetraspanin webs [21,22]. Membrane cholesterol contributes to the organization of these domains on the surface of live cells [23]. Cholesterol is also critical to many pathogens, including HCV [24] and *Plasmodium *infection [23]. Interestingly, it has been shown that CD81 is required for *Plasmodium *sporozoite entry and differentiation into hepatocytes [25,26]. Using a monoclonal antibody (mAb) that specifically recognizes a subset of mouse CD81 molecules associated with TEMs (MT81w), Silvie *et al*. have defined the role of TEM-associated CD81 in mice *Plasmodium *infection [23]. The similarities between *Plasmodium *and HCV liver infections indicate the importance of studying the role of TEM-associated CD81 in HCV infection.

In our study, infection of Huh-7 target cells with highly infectious HCVcc particles allowed us to isolate a cellular clone resistant to HCV infection which has lost CD81 expression (Huh-7w7 cells). We then took advantage of the emergence of these CD81-deficient cells to analyze the functionality of mCD81 in HCV infection and to study the role of TEM-associated CD81 in HCV infection.

## Results

### Generation of Huh-7 cells resistant to HCV infection

**Figure 1:**
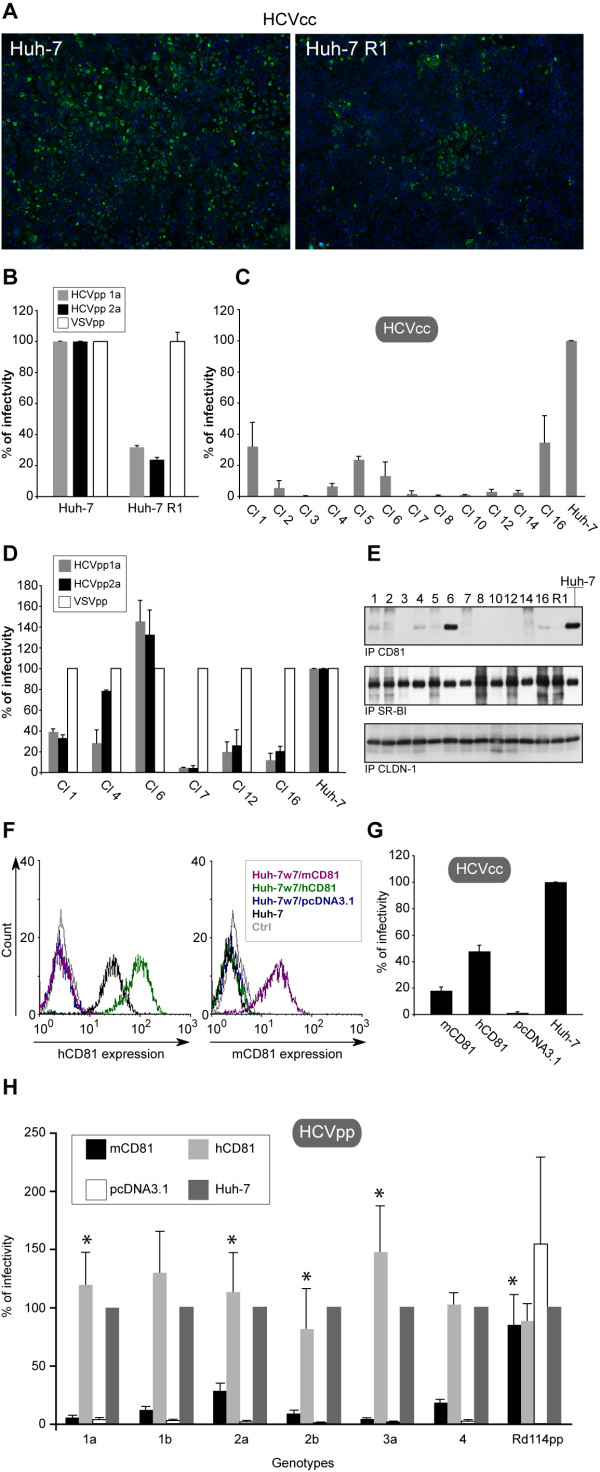
**Ectopic expression of CD81 in HCV-resistant Huh-7 cells restores HCV permissivity**. **A**, Huh-7 cells and R1 cell population infected with JFH-1 HCVcc were processed for double-label immunofluorescence for capsid protein (green) and nuclei (blue, Hoechst). **B**, Cells were infected with virus pseudotyped with HCV envelope proteins from 1a (HCVpp 1a) or 2a (HCVpp 2a) or VSV G envelope protein (VSVpp). **C**, Huh-7 cells and R1 individual cellular clones were infected with HCVcc expressing *Renilla *luciferase. In parallel, Huh-7 cells and some of the clones were infected with HCVpp 1a, HCVpp 2a or VSVpp (**D**). Results are presented as relative percentages to HCVcc (C) and HCVpp (D) infectivity on Huh-7 cells. HCVpp infections (D) were also normalized to VSVpp infections on Huh-7 cells. **E**, Surface biotinylated cell lysates were immunoprecipitated with anti-CD81 (5A6), anti-SR-BI (NB400-104H3) or anti-CLDN-1 (JAY.8) mAbs. Proteins were revealed by Western blotting with HRP-conjugated streptavidin. **F**, Flow cytometry analysis of CD81 cell surface expression. Cells were stained using an anti-hCD81 (1.3.3.22, left panel) or an anti-mCD81 (MT81, right panel), and secondary antibodies conjugated with PE. Ctrl corresponds to Huh-7 cells stained only with secondary antibodies. Cell lines were infected with HCVcc (**G**) and in parallel with HCVpp (**H**) generated with envelope proteins from different genotypes or virus pseudotyped with feline endogenous virus RD114 glycoprotein (Rd114pp). Results are presented as relative percentages to HCVcc (G) and HCVpp (H) infectivity on Huh-7 cells. *P *< 0.05 as calculated by the Mann-Whitney's test; *, statistically not significant difference in HCVpp entry compared to entry into Huh-7 cells.

In a previous study, we have shown that a HCV JFH-1 full-length RNA genome encoding mutations in the capsid and E2 sequences (JFH-1/CS-N6) releases highly infectious HCVcc particles which display a marked cytopathic effect on target cells [27]. Infection of Huh-7 cells with these particles led to the selection of few living cells that were resistant to HCV infection. In order to analyze the capacity of these cells to resist to HCVcc infection, they were amplified and treated with interferon α to eliminate any potential remaining virus. This cell population, called Resistant 1 (R1), displayed reduced levels of JFH-1 HCVcc infection compared to parental Huh-7 cells (Figure 1A). In parallel, we infected the R1 cell population with retroviral particles harboring HCV envelope glycoproteins of genotypes 1a or 2a (HCVpp-1a or HCVpp-2a, respectively) and found reduced levels of HCVpp infection in comparison to Huh-7 cells (Figure 1B). Both cell lines were not infected by particles devoid of envelope proteins (data not shown) and were equally infected with the positive control VSVpp, which infects virtually all type of cells (Figure 1B).

To further analyze this cellular resistance to HCV infection, cellular clones were isolated by limiting dilution and their sensitivity to HCVcc and HCVpp infection was analyzed. As shown in Figure 1C, several clones were resistant to infection (clones 3, 7, 8, 10, 12 and 14) while others exhibited some susceptibility to HCVcc (clones 1, 2, 4, 5, 6 and 16), but greatly reduced when compared to the parental Huh-7 cells. Interestingly, analysis of the sensitivity of several clones to HCVpp infection showed similar reduced infectivity levels (Figure 1D), indicating that the entry step of HCV life cycle is affected in these cells. The only major difference was observed for clone 6, which was barely permissive for JFH-1 infection but highly permissive for HCVpp, suggesting that replication or assembly of HCVcc is likely affected in these cells.

### Ectopic expression of human and mouse CD81 in resistant cells restores HCV permissivity

The HCV entry stage is a multistep process involving several cellular factors (reviewed in [9]). Among these molecules, the tetraspanin CD81, the Scavenger Receptor class B type I (SR-BI), and the tight junction protein claudin 1 (CLDN-1) play key roles. Since the absence of one of these molecules might explain the differences in infectivity of the R1 cell clones, their expression levels were examined (Figure 1E). Experiments of surface biotinylation followed by immunoprecipitations with specific mAbs showed that the cell surface expression levels of SR-BI and CLDN-1 were similar in each clone, whereas CD81 expression differed among the clones. CD81 cellular expression levels in R1 cell clones were also tested by anti-CD81 western-blotting over total cell lysates and similar results were obtained (data not shown). Interestingly, non permissive R1 cell clones were also negative for CD81 expression, indicating that HCV entry defect observed in clones 3, 7, 8, 10, 12 and 14 is likely due to the absence of CD81 expression.

To confirm our hypothesis, we ectopically expressed CD81 in one of the non-permissive Huh-7 R1 cell clones (clone 7) that we called Huh-7w7 cells. Plasmids expressing human CD81 (hCD81), mouse CD81 (mCD81) or empty expression vector (pcDNA3.1) were stably transfected in Huh-7w7 cells. The CD81 expression level was next controlled by flow cytometry analysis using 1.3.3.22 anti-hCD81 (Figure 1F, **left panel**) and MT81 anti-mCD81 (Figure 1F, **right panel**) mAbs. Cell surface expression of hCD81 in Huh-7w7/hCD81 cells was higher than in parental Huh-7 cells, whereas no hCD81 expression was detectable in Huh-7w7/pcDNA3.1 and Huh-7w7/mCD81 cells. mCD81 was also highly expressed in Huh-7w7/mCD81 cells (Figure 1F, **right panel**) and expression level was comparable with the one of Hepa1.6 cells that naturally express mouse CD81 (data not shown). Huh-7 cells and the complemented Huh-7w7 populations displayed similar expression levels of the control tetraspanin CD151 (data not shown).

We next tested the sensitivity of the different cell lines to HCVcc and HCVpp infection. Control cells expressing the empty vector pcDNA3.1 were totally resistant to HCV infection (Figures 1G and 1H). In contrast, Huh-7w7/hCD81 cells were equally or slightly more infected by HCVpp than parental Huh-7 cells (Figure 1H). Thus, ectopic expression of hCD81 fully restores permissivity to HCVpp harboring HCV envelope glycoproteins from different genotypes indicating that CD81 expression is likely the only defect for HCV entry in Huh-7w7 cells. However, the level of infectivity of Huh-7w7/hCD81 cells by HCVcc was 50%, as compared to the one of Huh-7 cells, indicating that despite being highly expressed, hCD81 did not fully restore permissivity to HCVcc. Overexpression of CD81 (Figure 1F) in Huh-7w7/hCD81 cells may lead CD81 to oligomerize, as shown for CD9 another tetraspanin [28], in less permissive CD81 molecules to HCVcc infection. The entry efficiency of HCVpp will not be affected in this context but only driven by CD81 expression levels. It has to be noted that differences in HCVcc and HCVpp entries have already been shown [29].

Interestingly, ectopic expression of mCD81 in Huh-7w7 cells was also able to restore HCV permissivity. As shown in Figure 1G, the level of permissivity to HCVcc of Huh-7w7/mCD81 cells was 20% of the one of parental Huh-7 cells. In addition, permissivity of Huh-7w7/mCD81 cells to HCVpp bearing glycoproteins from different genotypes was analyzed and showed that mCD81 supports infection with HCVpp from genotypes 2a and 4, with 29% and 19% of level of infectivity respectively, as compared to the one of Huh-7 cells (Figure 1H). In contrast to Flint *et al*. [15], we did not observe any significant infectivity for HCVpp harboring glycoproteins from genotypes 1a and 1b.

**Figure 2:**
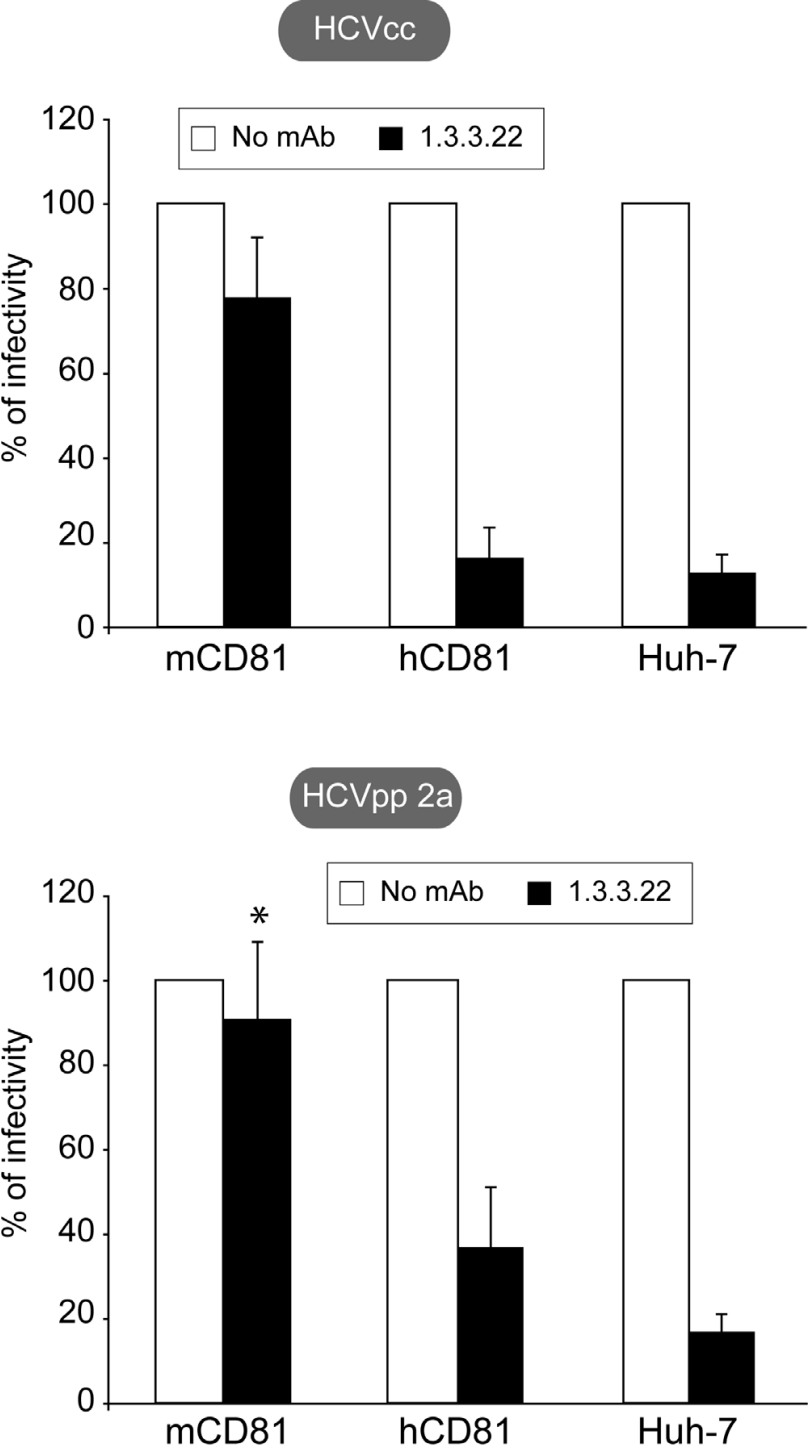
**Anti-hCD81 mAb inhibits HCV infection of hCD81 expressing cells but not of Huh-7w7/mCD81 cells**. HCVcc (upper panel) and HCVpp 2a (lower panel) infections of cell lines were performed in absence (white histograms) or presence (black histograms) of 1.3.3.22 anti-hCD81 mAb (3 μg/ml). At 2 days post-infection, cells were lysed and processed as described in methods. *P *< 0.05 as calculated by the Mann-Whitney's test; *, statistically not significant difference in HCVcc infectivity compared to infectivity in absence of antibodies.

It is worth noting that the sensitivity of Huh-7w7/mCD81 cells to HCV infection is solely due to the expression of mCD81 since anti-hCD81 mAbs (1.3.3.22; Figure 2 and 5A6; not shown) efficiently inhibited HCVcc and HCVpp infection of Huh-7 and Huh-7w7/hCD81 cells, but did not significantly affect the infectivity of Huh-7w7/mCD81 cells. These results indicate that no residual expression of hCD81 is responsible for permissivity since in such a case infection would be fully inhibited by the anti-hCD81 mAbs. Control experiment performed with irrelevant antibodies did not inhibit HCV infectivity (data not shown).

Taken together, these data indicate that HCV infection is directly related to CD81 expression in Huh-7w7 cells. Most importantly, mCD81 in the context of such human hepatocytes is able to some extent to mimic the role of hCD81 in HCV entry and likely interacts in a similar way with cellular factors.

### Role of TEM-associated CD81 in HCV entry

Tetraspanins associate extensively with each other and other membrane proteins to form the TEMs. Recently, Silvie *et al*. have described the MT81w mAb, which specifically recognizes mouse CD81 associated with other tetraspanins. This is evidenced by the lack of recognition of CD81 after cell lysis with detergents that do not preserve tetraspanin-tetraspanin interactions, and by the complete removal of the CD81 pool recognized by MT81w following immunodepletion of tetraspanin complexes [23]. CD81 is required for invasion of hepatocytes by sporozoites of human malaria *Plasmodium falciparum *and rodent malaria *Plasmodium yoelii *parasites [26]. Using MT81w antibody, Silvie *et al*. have shown that the subset of CD81 associated with TEMs contributes to *Plasmodium *sporozoite infection [23]. Such an antibody preferentially recognizing human CD81 associated with TEMs is not available. However, since Huh-7w7/mCD81 cells are susceptible to HCVcc and HCVpp-2a infection, we next took advantage of this model and the MT81w mAb to study the role of TEM-associated CD81 in the early steps of HCV life cycle.

**Figure 3:**
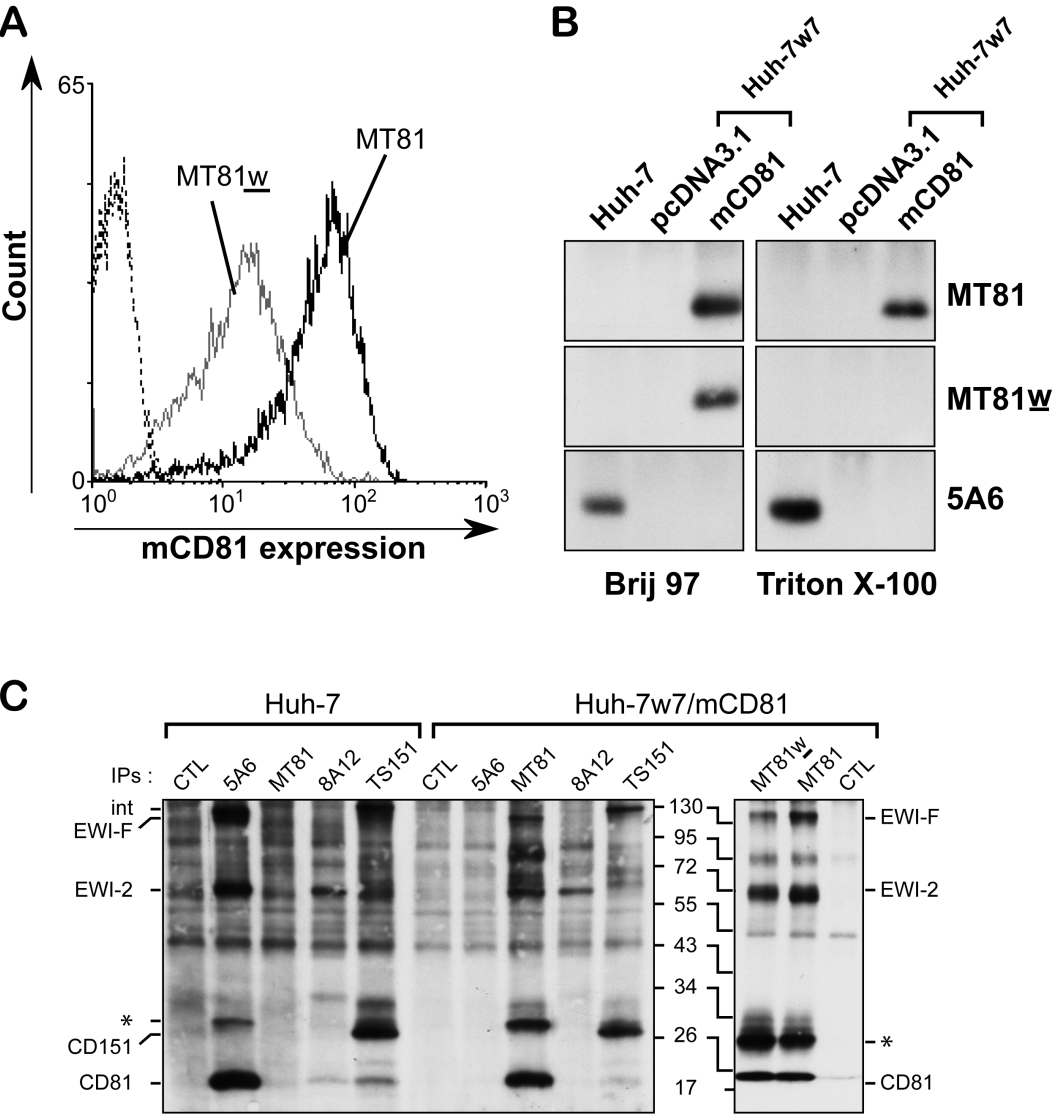
**Recognition of TEM-associated CD81 in Huh-7w7/mCD81 cells**. **A**, Flow cytometry analysis of Huh-7w7/mCD81 cells stained with the mAbs MT81 and MT81w. Cells stained only with PE-conjugated secondary antibody were used as control (dotted line). **B**, Cell lines were surface biotinylated and lysed in the presence of Brij97 or Triton X-100 before immunoprecipitation with MT81, MT81w and 5A6 mAbs. Immunoprecipitates were revealed by western blotting using peroxidase-conjugated streptavidin. **C**, Cell lines were surface biotinylated and lysed in buffer containing 1% Brij97 and divalent ions. Immunoprecipitations were then performed with 5A6, MT81, MT81w, 8A12 (anti-EWI-2), TS151 (anti-CD151) or irrelevant (CTL) mAbs. Immunoprecipitates were revealed by western blotting using peroxidase-conjugated streptavidin. The molecular weights of the prestained molecular ladders are indicated in KDa. The asterisks indicate dimers of CD81.

Using the MT81w anti-mCD81 mAb, we first characterized the subpopulation of mCD81 that is associated with TEMs on the cell surface of Huh-7w7/mCD81 cells (Figure 3A). As shown by flow cytometry analysis, the intensity of MT81w labeling only reached 32 ± 14%, depending on the experiment, of the intensity with MT81 in Huh-7w7/mCD81 cells, indicating that only a fraction of CD81 molecules is engaged in tetraspanin microdomains on these cells, as described for Hepa1–6 cells [23]. However, we cannot exclude that the lower affinity of MT81w may lead to an underestimate of the ratio of CD81 engaged in TEMs. The specificity of MT81w to recognize TEM-associated CD81 in Huh-7w7/mCD81 cells was confirmed by immunoprecipitation experiments from biotinylated cell lysates made under different detergent conditions. Tetraspanin microdomains are typically disrupted by Triton X-100, but are retained in less hydrophobic detergents such as Brij 97 [30]. As shown in Figure 3B, 5A6 and MT81 mAbs precipitated hCD81 and mCD81, respectively, under both detergent condition. In contrast, MT81w was able to precipitate mCD81 only from Brij97 lysates preserving tetraspanin-tetraspanin interactions, but not from Triton X-100 lysates. These results show that expression of mCD81 in Huh-7w7 cells allowed to reconstitute tetraspanin webs that are specifically recognized by the well characterized MT81w mAb [23].

To ensure that similar molecular web interactions occur in Huh-7w7/mCD81 and Huh-7 cells, we next analyzed TEM composition in immunoprecipitation experiments of surface biotinylated cell lysates. Since lysis in Brij 97 preserves tetraspanin-tetraspanin interactions, any anti-tetraspanin mAb can co-immunoprecipitate the entire set of proteins present in tetraspanin microdomains [31]. The tetraspanin pattern obtained with Huh-7 cells using 5A6 hCD81 mAb is shown in Figure 3C. The major proteins co-immunoprecipitated with CD81 have an apparent molecular mass consistent with that of EWI-2 and EWI-F, two major partners of CD81 [30,32,33]. The identity of these proteins was confirmed by direct immunoprecipitation (Figure 3C and data not shown), as previously described [19]. Interestingly, MT81 and MT81w immunoprecipitations of mCD81 in Huh-7w7/mCD81 cells gave a pattern similar to that of hCD81 in Huh-7 cells (Figure 3C). EWI-2 and EWI-F proteins were co-immunoprecipitated with mCD81 in Huh-7w7/mCD81 cells. In addition, immunoprecipitation with an anti-CD151, another tetraspanin, co-immunoprecipitated a fraction of mCD81 in Huh-7w7/mCD81 cells as well as hCD81 in Huh-7 cells (Figure 3C, lines TS151). Altogether, in spite of slight differences in stoichiometry, these results show that mCD81 in Huh-7w7/mCD81 cells is engaged in similar web interactions than hCD81 in Huh-7 cells.

**Figure 4:**
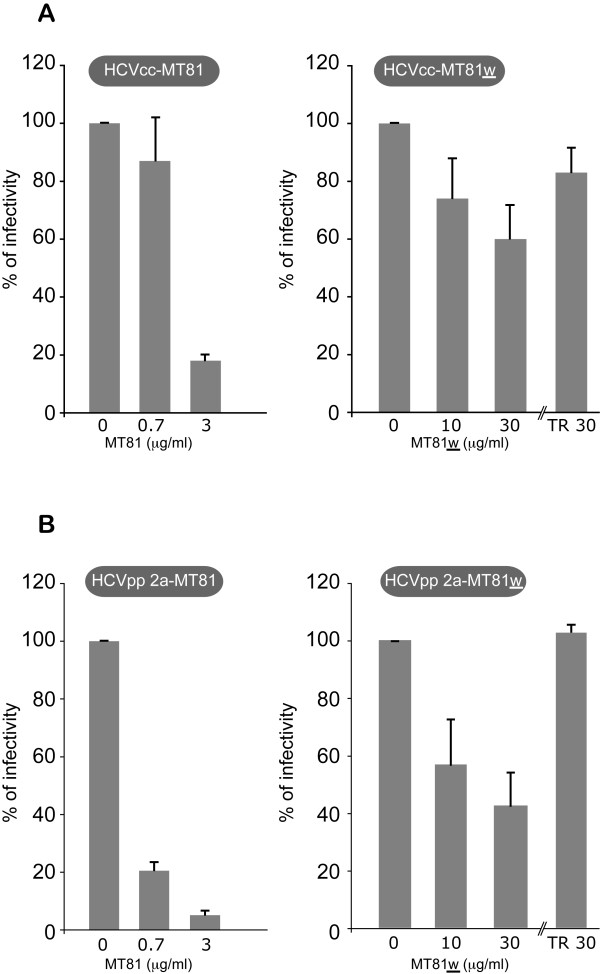
**Neutralization assay of HCV infection with MT81 and MT81w antibodies**. HCVcc (**A**) and HCVpp 2a (**B**) infections of Huh-7w7/mCD81 cells were performed in presence of indicated concentrations of MT81 (left panels) or MT81w (right panels) mAbs or in presence of 30 μg/ml of transferrin receptor (right panels, TR30). At 2 days post-infection, cells were lysed and processed as described in methods. *P *< 0.05 as calculated by the Mann-Whitney's test.

We then analyzed the ability of MT81 and MT81w to inhibit HCVcc and HCVpp infectivity. As shown in Figures 4A and 4B, MT81 mAb, which recognizes the whole population of CD81, efficiently inhibited both HCVcc infection and HCVpp entry into Huh-7w7/mCD81 cells. Indeed, MT81 inhibited 80% of HCVcc infection and 95% of HCVpp infection at low concentrations (3 μg/ml). In contrast, MT81w was poorly neutralizing since it only induced an inhibition of 40% and 60% of HCVcc and HCVpp infection, respectively, at tenfold higher concentrations (30 μg/ml). However, it has to be noted that MT81w mAb might be a low-affinity antibody, as compared to MT81 [23]. The specificity of the observed inhibitory effect was ensured by using an irrelevant antibody at the highest concentration (anti-transferrin receptor antibody CD71 at 30 μg/ml, Figure 4 TR30). As expected, MT81 and MT81w did not affect HCVcc or HCVpp infectivity levels of Huh-7 cells (data not shown).

Together, our results suggest that TEM-associated CD81 molecules might not play a central role in HCV entry. However, since we cannot exclude a partial recognition of TEM-associated CD81 molecules by the low affinity MT81w mAb or that the epitope recognized by this antibody is located outside of the E2 binding region, we further analyzed the role of TEM-associated CD81 in HCV entry using other approaches.

### Role of cholesterol in HCV infection and the association of CD81 with TEM

**Figure 5:**
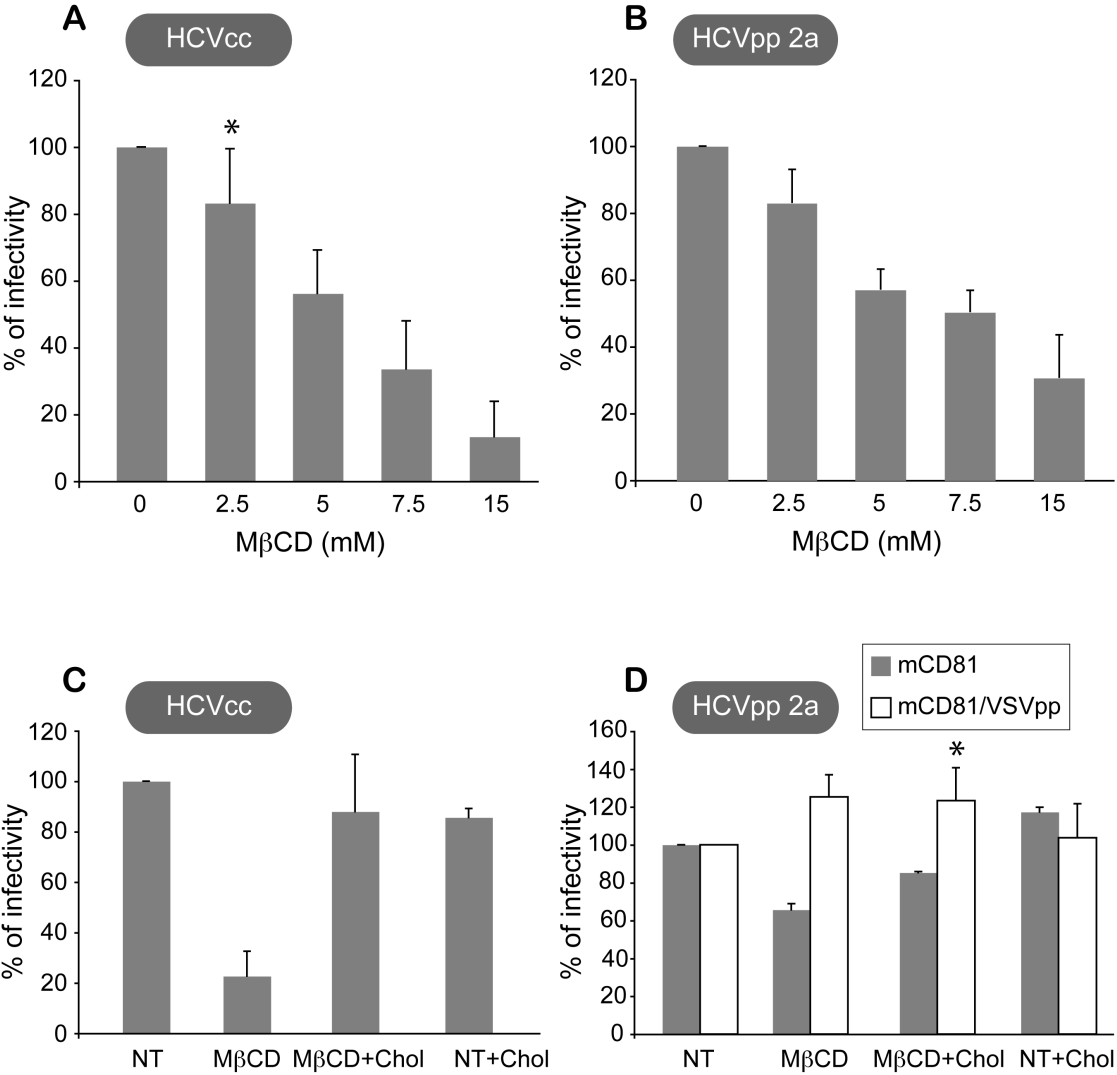
**Depletion of cellular cholesterol decreases HCV infection of Huh-7w7/mCD81 cells**. Huh-7w7/mCD81 cells were pretreated with increasing concentrations of MβCD prior to infection with HCVcc (**A**) or HCVpp 2a (**B**). Huh-7w7/mCD81 cells were untreated (NT) or pretreated with 7.5 mM of MβCD (MβCD) and then treated or not with 2.5 mM of preformed MβCD-Cholesterol complexes (Chol) (C and D). After treatment, cells were infected with HCVcc (C) or HCVpp-2a or VSVpp (D). At 2 days post-infection, cells were lysed and processed as described in methods. *P *< 0.05 as calculated by the Mann-Whitney's test; *, statistically not significant difference in HCV infectivity compared to infectivity in absence of drugs.

Cellular cholesterol has been shown to modulate the organization of tetraspanin microdomains [23] and to be involved in HCV life cycle [34]. To further analyze the role of TEM-associated CD81 in HCV infection, we next assessed the effect of cholesterol depletion on HCV infection. Huh-7w7/mCD81 cells were treated with increasing amounts of methyl-beta-cyclodextrin (MβCD), a cyclic oligosaccharide that selectively removes cholesterol from the plasma membrane without incorporating into the membrane [35]. Treatment of Huh-7w7/mCD81 cells with MβCD prior to infection resulted in a dose-dependent inhibition of HCVcc (Figure 5A) and HCVpp-2a (Figure 5B) infectivity. In both set of experiments the maximal inhibition of HCV infection was reached at an MβCD concentration of 15 mM, which decreased the cellular cholesterol content by fivefold (data not shown). Moreover, inhibition of infection was specifically due to cholesterol removal from the cell surface, since it was reversed by cholesterol replenishment with MβCD-cholesterol complexes before HCV infection (Figures 5C and 5D). Such preformed MβCD-cholesterol complexes are known to replenish cells with cholesterol [36]. It has to be noted that MβCD treatment had no effect on VSVpp entry (Figure 5D), which is clathrin dependent, indicating that HCVpp entry inhibition was not due to disruption of clathrin-enriched domains following cholesterol depletion [37-39]. In addition, cell treatment with MβCD at 15 mM three hours after cell/virus contact did not have any effect on infection (data not shown), indicating that membrane cholesterol is required at the entry step and MβCD is not toxic under our experimental conditions. Cholesterol depletion and replenishment experiments were performed on Huh-7 cells and gave similar results (data not shown).

Altogether, our data confirm the role of cholesterol in HCV entry and bring to light a similar response of Huh-7w7/mCD81 and Huh-7 cells to cholesterol depletion and replenishment in terms of HCV infection.

**Figure 6:**
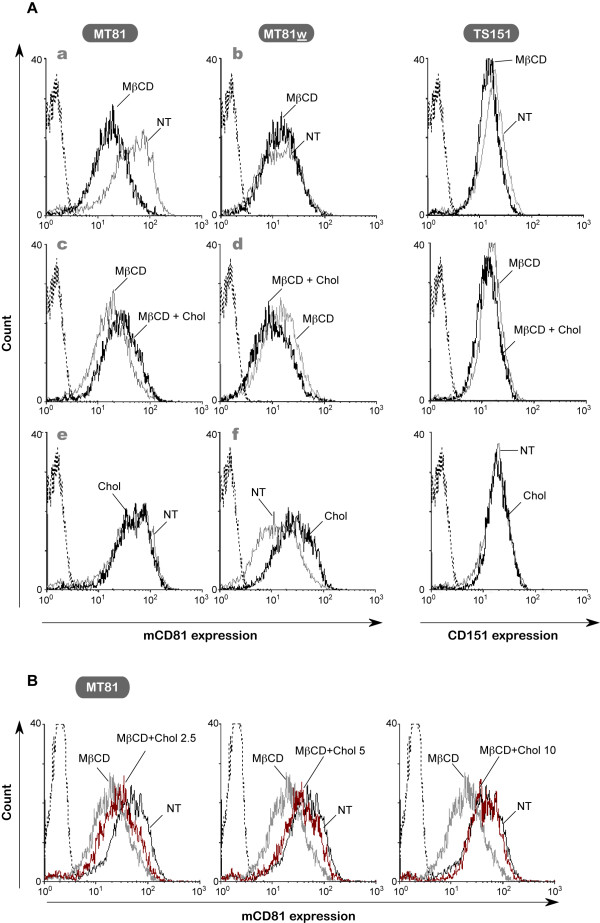
**Cholesterol depletion affects total CD81 cell surface expression**. **A**, Flow cytometry analysis of CD81 and CD151 expression on the cell surface of Huh-7w7/mCD81 cells. Upper panels: cells were treated with 7.5 mM of MβCD (MβCD) or left untreated (NT). Middle panels: cells were treated with 7.5 mM of MβCD (MβCD) followed by 2.5 mM of MβCD-Cholesterol (MβCD + Chol). Lower panels: cells were treated with 2.5 mM of MβCD-Cholesterol (Chol) or left untreated (NT). **B**, Cells were treated with 7.5 mM of MβCD (MβCD) followed by increasing concentrations (in mM) of MβCD-Cholesterol (MβCD + Chol) and total cell surface CD81 expression compared to untreated cells (NT) was measured using MT81 mAb.

We next analyzed by flow cytometry the surface expression of CD81 and its association with TEMs in Huh-7w7/mCD81 cells treated with MβCD or MβCD-cholesterol complexes (Figure 6), and expression of CD151 was used as a control (right panels). MβCD treatment of Huh-7w7/mCD81 cells reduced MT81 labelling by 58 ± 7% (Figure 6Aa), suggesting that cholesterol depletion induced a decrease in total cell surface expression of mCD81 in Huh-7w7/mCD81 cells. Even with cholesterol replenishment, CD81 expression level could not be restored to conditions that would enable HCV infectivity (Figure 6Ac, MβCD+Chol). Incubation of MβCD-treated cells with increasing concentrations of preformed MβCD-cholesterol complexes raised cell surface mCD81 expression level (Figure 6B). However, a concentration four times higher than needed to reverse the inhibitory effect of MβCD on HCV infectivity (10 mM instead of 2,5 mM) was necessary to reach the cell surface mCD81 expression level of untreated cells. Interestingly, treatment with MβCD alone had no effect on TEM-associated mCD81 population in Huh-7w7/mCD81 cells, as determined using MT81w (Figure 6Ab). Conversely, cholesterol enrichment of non depleted cells with preformed MβCD-cholesterol complexes led to a 2 ± 0.6 fold increase of TEM-associated mCD81 population (Figure 6Af), without any change in the total CD81 population (Figure 6Ae). These results confirm the role of cholesterol in TEM organization. Expression of CD151 under different conditions was not affected (Figure 6A, **right panels)**.

Our results differ from those of Silvie *et al*. showing that similar MβCD treatment of Hepa1–6 cells did not lead to a significant decrease of total CD81 cell surface expression [23]. However, it has to be noted that the tetraspanin CD9, expressed in Hepa1–6 cells but not in Huh-7 cells, has been shown to increase stability of tetraspanin complexes [40].

In conclusion, cellular cholesterol depletion mediated by MβCD strongly affects HCV infection, but it has no effect on TEM-associated mCD81 in Huh-7w7/mCD81 cells. Again, these data do not support a key role for TEM-associated CD81 molecules in HCV infection.

**Figure 7:**
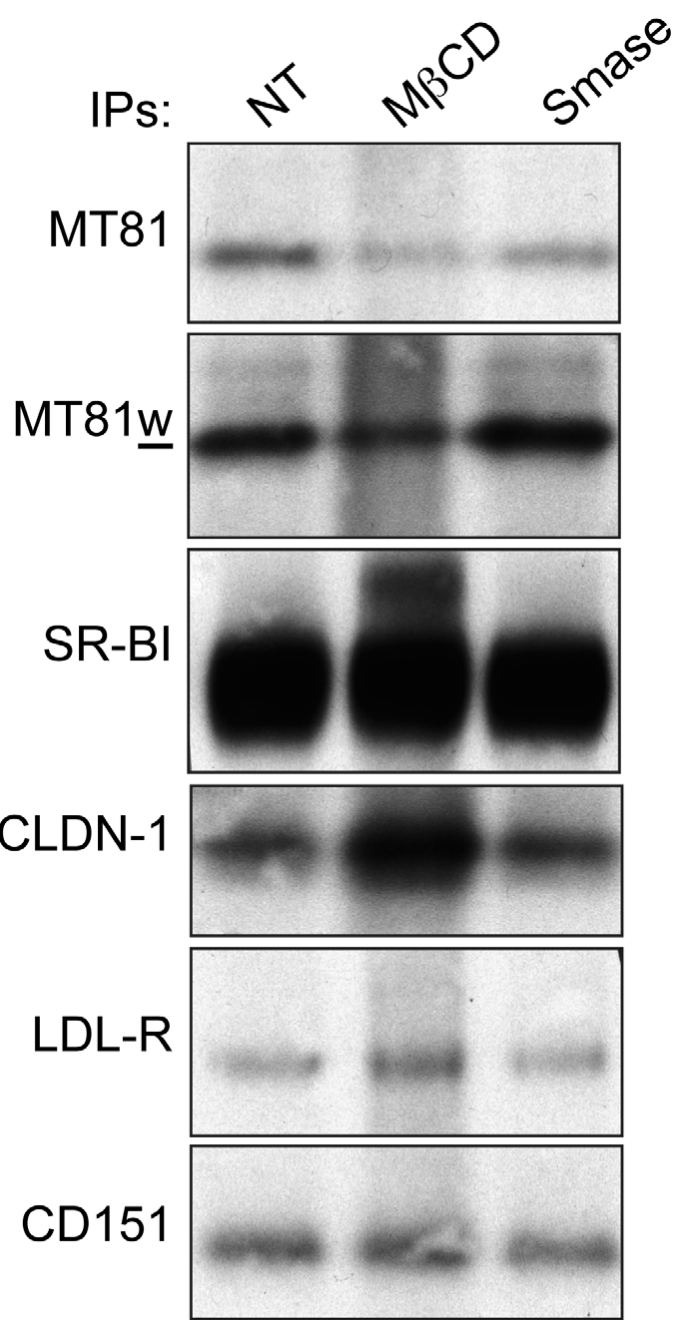
**Cholesterol depletion and ceramide enrichment do not reduce cell surface expression of other HCV entry factors**. Huh-7w7/mCD81 cells were treated with 7.5 mM MβCD, with 0.5 unit Smase/ml or left untreated (NT) 30 min at 37°C. Cells were then surface biotinylated and lysed in buffer containing 1% Brij97 and divalent ions. Immunoprecipitations were performed with indicated mAbs. Immunoprecipitates were revealed by western blotting using peroxidase-conjugated streptavidin.

Finally, we ensured that MβCD-induced inhibition of HCV entry did not lead to a reduced expression level of another HCV entry factor. We analyzed the expression levels of SR-BI, CLDN-1 and LDL receptor (LDL-R), three other major actors of HCV entry [9]. The tetraspanin CD151 was used again as a control. Since no antibody against extracellular loops of CLDN-1 is available for flow cytometry, we performed our analyses by immunoprecipitation of surface biotinylated cell lysates. As shown above, treatment of Huh-7w7/mCD81 cells with MβCD was accompanied by a reduced expression level of CD81, as detected by MT81 (Figure 7). We also found a reduced immunoprecipitation of CD81 by MT81w after MβCD treatment. Cholesterol depletion prior to lysis in Brij 97 likely led to a harsher effect of the detergent leading to a partial dissociation of tetraspanin complexes recognized by MT81w. Interestingly, treatment of Huh-7w7/mCD81 cells with MβCD did not lead to a reduction of cell surface expression of SR-BI, CLDN-1 or LDL-R. It has to be noted that SR-BI and CLDN-1 seemed even more expressed after MβCD treatment (Figure 7). This increased expression of SR-BI after MβCD treatment was confirmed by flow cytometry (data not shown) and has been previously described by others [24,41-43]. It has also been suggested that CLDN-1 might be in membrane domains resistant to MβCD treatment [44,45]. Altogether, our results show that MβCD-induced inhibition of HCV entry was solely due to reduced levels of cholesterol and CD81.

### Role of ceramide in TEM-associated CD81 and in HCV infection

**Figure 8:**
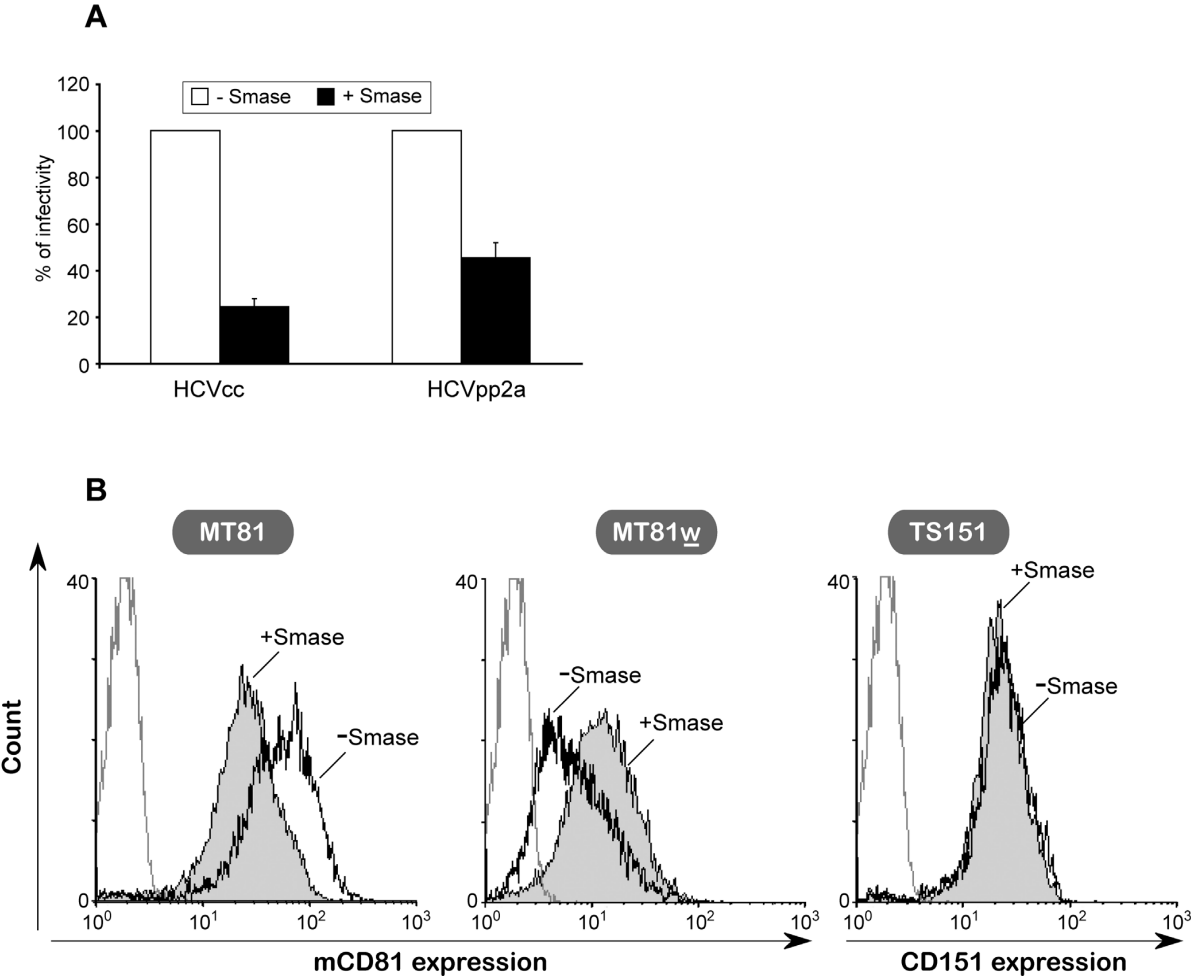
**Ceramide enrichment of the plasma membrane of Huh-7w7/mCD81 cells inhibits HCV entry and increases association of CD81 with TEMs**. **A**, Huh-7w7/mCD81 cells were pretreated (+Smase) or not (-Smase) with Smase prior to infection with HCVcc or HCVpp 2a. At 2 days post-infection, cells were lysed and processed as described in methods. *P *< 0.05 as calculated by the Mann-Whitney's test. **B**, Huh-7w7/mCD81 cells pretreated (+Smase) or not (-Smase) with Smase were stained with MT81 (left panel), MT81w (middle panel) or TS151 (right panel) mAbs. Cells stained only with PE-conjugated secondary antibody were used as control (dotted line).

Beyond cholesterol, sphingolipids are also known to be important for the organization of the plasma membrane. Among them, sphingomyelin can be converted into ceramide by sphingomyelinase (Smase), and increasing ceramide concentration can lead to lipid microdomain reorganization [46]. We have previously reported that ceramide enrichment of the plasma membrane of Huh-7 cells following sphingomyelin hydrolysis by sphingomyelinase strongly inhibits HCV entry and reduces CD81 cell surface expression level by 50% [47]. Since sphingomyelin influences CD81 cell surface expression as well as HCV infection, we sought to determine the effect of the Smase treatment on TEM-associated CD81 population. Huh-7w7/mCD81 cells were pre-treated with Smase, washed and then infected with HCVcc or HCVpp-2a. Similarly to Huh-7 cells, Huh-7w7/mCD81 cells were affected by Smase treatment, resulting in 70–80% and 50–60% inhibition of HCVcc and HCVpp-2a infection, respectively (Figure 8A).

In order to determine whether these inhibitions were associated with changes in cell surface expression of CD81, we analyzed by flow cytometry the CD81 surface expression level after Smase treatment (Figure 8B). Interestingly, Smase treatment of Huh-7w7/mCD81 cells led to a significant reduction (52 ± 18%) in MT81 labelling and conversely to significant increase (277 ± 74%) in MT81w labelling, indicating that the treatment induced a reduction of total mCD81 expression and an increased association of CD81 with TEM. As expected, Smase treatment did not affect the expression of the control tetraspanin CD151 (Figure 8B).

We next ensured that Smase-induced inhibition of HCV entry was not also associated with reduced expression level of another HCV entry factor. As described above, we analyzed the expression levels of SR-BI, CLDN-1 and LDL-R after treatment of Huh-7w7/mCD81 cells with Smase. As shown above (Figure 8B), treatment with Smase was accompanied by a reduced expression level of CD81, as detected by MT81 (Figure 7). In accordance with our previous results (Figure 8B), we also found an increased immunoprecipitation of CD81 by MT81w after Smase treatment. Interestingly, expression level of SR-BI, CLDN-1 or LDL-R were not affected following treatment of cells with Smase (Figure 7).

Thus, Smase treatment of Huh-7w7/mCD81 cells resulted in HCV entry inhibition and increase of TEM-associated mCD81 population. In agreement with previous data, these results indicate that TEM-associated CD81 does not play a major role in HCV entry. Smase treatment resulted also in a significant decrease of cell surface expression of CD81 on Huh-7 cells (data not shown), as described previously [47]. The similarity of Huh-7 and Huh-7w7/mCD81 cells responses to Smase treatment tends to show that results obtained with Huh-7w7/mCD81 cells can be extrapolated to Huh-7 cells.

## Discussion and conclusion

We have previously shown that mutations (CS-N6) in the structural proteins of the JFH-1 genome increase the production and infectivity of HCVcc particles, leading to an accelerated cytopathic effect [27]. Interestingly, infection of Huh-7 cells with such particles led us to isolate cellular clones exhibiting different levels of permissivity to HCVcc and HCVpp. For most of them, reduced HCV infection levels were directly related to their reduced expression level of CD81, while other entry molecules such as SR-BI and CLDN-1 were not modified. Our observation is in accordance with previously published data [29,48-50]. Ectopic expression of CD81 in Huh-7w7 cells, one of the resistant cell clones, restored HCV permissivity indicating that CD81 deficiency alone was responsible for the resistance to HCV infection in these cells. In agreement with previous studies [29,48,51], we did not observe any variation in HCV genome replication in Huh-7w7 cells in comparison to Huh-7 cells (data not shown), suggesting that CD81 is not involved in this step of the viral cycle. Masciopinto *et al*. showed that CD81 and HCV envelope glycoproteins could be detected in exosomes of mammalian cells, suggesting that HCV may intracellularly interact with CD81 allowing its export [52]. They pointed out a possible role of CD81 in assembly and release of HCV particles. However, our results indicate that CD81 does not participate to HCV assembly or release of new viral particles, since the supernatant of Huh-7w7 cells transfected with full-length HCV RNA infected naïve Huh-7 cells to a level comparable to that of the supernatant from transfected Huh-7 cells. Thus, Huh-7w7 cells constitute a new tool allowing to investigate the involvement of CD81 in HCV entry and offering a new single-cycle replication system, as already used by others [29].

The molecular determinants of HCV-CD81 interaction have been analyzed by several groups by using biochemical assays (reviewed in [53]). However, Flint *et al *have highlighted the limitation of these approaches since various mutated CD81 sequences previously reported to abrogate E2-CD81 interaction, were able to restore permissivity in HepG2 cells [15]. In our study, we show that ectopic expression of human and mouse CD81 proteins in human hepatoma cells devoid of CD81 conferred susceptibility to infection by HCVcc and HCVpp at various levels. Interestingly, mCD81 protein supports infection by HCVcc and HCVpp bearing glycoproteins from genotypes 2a and 4 suggesting that, in accordance with other studies [15,17], CD81 is not the sole determinant of species susceptibility to HCV. Other additional cellular factors likely modulate HCV entry. In addition, interaction/organization levels and stoichiometry between entry factors and plasma membrane lipids may regulate species susceptibility to HCV.

CD81 belongs to the tetraspanin family of which members have the distinctive feature of clustering dynamically with numerous partner proteins and with one another in membrane microdomains. Within this web, the associations of tetraspanins with their nontetraspanin partner molecules have been referred to as primary, and tetraspanin can interact with each other through their associated partner [12]. In contrast to primary complexes, tetraspanin-tetraspanin interactions are not stoichiometric and palmitoylation is necessary for the maintenance of these interactions [28,40,54,55]. It is still unknown whether all tetraspanins expressed in a certain cell are associated with each other. Importantly, tetraspanins associate indirectly with additional proteins. Functionally, these interactions cluster in TEM, enabling lateral dynamic organization in the membrane and the cross-talk with intracellular signalling and cytoskeletal structures [21]. In our study, generation of a human cell line expressing mCD81 (Huh-7w7/mCD81 cells) permissive to HCV infection allowed us to analyze the role of TEM-associated CD81 in HCV infection. This study could be performed with two recently described mAbs: MT81, which recognizes total mCD81; and MT81w, which specifically recognizes a fraction of mCD81 associated with other tetraspanins [23]. It is worth noting that such a tool allowing the detection of hCD81 associated with TEMs is not available. We first determined the inhibitory effect of both mAbs on HCVcc and HCVpp infection: MT81 strongly inhibited HCV infection, whereas MT81w led to a weak inhibition of infection at saturing concentrations. This reduced capacity of MT81w mAb to inhibit HCV infection suggests that TEM-associated CD81 molecules, recognized by this mAb, are not the exclusive site of infection. In accordance with these results, ceramide enrichment of plasma membrane leading to an increased association of CD81 with TEMs highly inhibits HCV infection. While palmitoylation is not the only mechanism by which tetraspanins interact with each other, it has been shown to play an essential role in TEM organization [28,40,54,55]. The ability of palmitoylation-defective CD81 to support infection by HCVpp [10] is again consistent with a minor role of TEM-associated CD81 in HCV entry. We cannot exclude that the epitope recognized by MT81w mAb on CD81 is not involved in HCV interaction. The partial inhibition of MT81w might also be the reflect of a partial recognition of the TEM-associated CD81 fraction, as previously suggested by Silvie et al. [23].

The entire HCV life cycle is associated with cholesterol metabolism in host cells (reviewed in [34]), and lipid composition of the plasma membrane seems very important for the HCV entry step. In our study, we showed that cholesterol depletion by treatment with MβCD strongly reduced HCV entry into target cells, and conversely cholesterol replenishment by MβCD-cholesterol complexes restored the infection levels. These results point out again the importance of cell membrane cholesterol in HCV entry, likely in the fusion process as has been previously suggested [56]. Very recently, we have shown that increasing the levels of ceramide in the plasma membrane induce a massive endocytosis of CD81 leading to a strong inhibition of HCV infection [47]. Interestingly, in the present study, we showed that following Smase treatment of Huh-7w7/mCD81 cells, expression of CD81 is inversely related to association of CD81 with tetraspanin webs. These results suggest that ceramide might specifically modify the levels of interaction or the cell surface distribution of TEM. In this regard, it has been shown that gangliosides play an important role in the organization of CD82-enriched microdomains [57]. Ceramide enrichment may also induce clustering of CD81 leading to an increased binding of MT81w mAb. In accordance with this hypothesis, it has been shown that high levels of ceramide induce large-scale clustering/capping of death receptors (e.g. Fas/CD95) required to initiate efficient formation of death-induced signalling complex [58,59]. Alternatively, MT81w may recognize an epitope of CD81 that is more exposed following ceramide enrichment. Further analyses are necessary to evaluate these hypotheses.

HCV and *Plasmodium *are two major pathogens targeting the liver. Both use the glycosaminoglycans for their initial attachment on the surface of hepatocytes [11,60-64], and lipidic transfer properties of scavenger receptor class B type I regulate infection of both pathogens [9,65,66]. CD81 is required for HCV and *Plasmodium *life cycle. Antibodies to CD81 or CD81 silencing strongly reduce the infection of hepatic cells and CD81-deficient mouse hepatocytes are resistant to infection by *Plasmodium *[26]. Using CD81/CD9 chimeras, it has been recently shown that CD81 LEL plays a critical role in sporozoite infection and a stretch of 21 amino acids is sufficient to confer susceptibility to infection [66]. In contrast to HCV infection, it seems that CD81 does not act directly as a receptor but is rather involved indirectly, likely by modulating the activity of an associated protein. This hypothesis is supported by the fact that CD81 associated to multiple proteins in the tetraspanin web plays a major role in sporozoite infection, since modulation of cellular cholesterol levels, which changes tetraspanin microdomain organization, has been shown to also modify the extent of CD81-dependent sporozoite infection [23]. In contrast, in our study, we demonstrated that TEM-associated CD81 is not used by HCV, indicating that these two pathogens, while using the same molecules, invade their host by different mechanisms.

## Methods

### Antibodies

5A6 (anti-CD81 kindly provided by S. Levy); ACAP27 (anti-HCV core, kindly provided by JF Delagneau); MT81 (anti-CD81), MT81w (anti-TEM associated CD81), 8A12 (anti-EWI-2) and TS151 (anti-CD151) mAbs were used in this study. The anti-Claudin-1 (JAY.8) was from Zymed, the anti-SR-BI (NB400-104H3) was from Novus, the anti-LDL receptor was from Progen, the anti-transferrin receptor antibody was from Biolegend (Ozyme) and the anti-hCD81 (1.3.3.22) was from Santa Cruz Biotechnology. Alexa^488^-conjugated goat anti-mouse was from Jackson Immunoresearch.

### Production of HCVcc and infection assays

Production of HCVcc and infection assays were performed as described [19]. To generate HCVcc expressing *Renilla *luciferase, we used the FL-J6/JFH-5'C19Rluc2AUbi genome [67] kindly provided by C.M. Rice (The Rockfeller University, New York). We replaced the region encoding the J6/JFH-1 HCV polyprotein with the CS-N6 JFH-1 sequence [27]. HCVcc were produced as previously described [7,27,67]. HCVcc were added to Huh-7 cells seeded the day before and incubated for 2 h at 37°C. The supernatants were then removed and the cells were incubated in DMEM 10% FBS at 37°C. At 40–48 h post-infection, cells were lysed and processed to measure the *Renilla *luciferase activities as indicated by the manufacturer (Promega). Luciferase activities were normalized for protein concentration in each cell lysate. In each figure, results are reported as the mean ± S.D. of three independent experiments.

### Generation of R1 cell population and resistant cellular clones

Huh-7 cells were infected (m.o.i. = 1) with JFH-1/I2/CS-N6 particles [27] 4 h at 37°C and then maintained for several weeks. Survival cells were amplified and treated with 200 UI/ml of IFN α. After six successive treatments with IFN α, cells were analysed by immunofluorescence and western blotting and subcloned by limiting dilution. The cells were seeded in 96-well plates at 1 cell/well in DMEM 10% FCS. Individual cell clones were amplified and named Huh-7w with a number corresponding to the clone.

### Cell transfection

Huh-7w7 cells were transfected using ExGen500 (Eurogentec) with plasmids expressing human CD81 (pcDNA3.1/hCD81), murine CD81 (pcDNA3.1/mCD81) [30] or the empty vector. Polyclonal populations were obtained by selection for 4 weeks with 600 μg/ml of Neomycin (Invitrogen).

### Antibody neutralization assay

Neutralization assays were performed by co-incubating HCVcc/HCVpp and antibodies with target cells 3 h at 37°C. Cells were further incubated for 48 h with DMEM 10% FCS before measuring the luciferase activities.

### Cholesterol depletion/replenishment and sphingomyelinase (Smase) treatment

Cholesterol depletion was carried out by incubating cells with different concentrations of methyl-β-cyclodextrin (MβCD, Sigma) in serum-free medium at 37°C for 20 min. Cholesterol replenishment of cholesterol-depleted cells was achieved by incubating cells with 1:10 (mol/mol) complex of cholesterol and MβCD (cholesterol water soluble, Sigma) using a 2.5 mM final cholesterol concentration in serum-free medium at 37°C for 15 min. Cholesterol levels in MβCD-treated cells were determined using Amplex Red Cholesterol Assay kit (Molecular Probes). Smase treatments were performed as previously described [47].

### Production of HCVpp and infection assays

HCVpp were produced as described previously [3,68] with plasmids kindly provided by B. Bartosch and F.L. Cosset (INSERM U412, Lyon, France). The plasmids encoding HCV envelope glycoproteins of genotypes 1b (UKN1B-5.23), 2b (UKN2B-1.1), 3a (UKN3A-1.28) and 4 (UKN4-11.1) were kindly provided by J. Ball (Nottingham University, UK) [69]. The genotype 1a plasmid (strain H) has been described previously [3] and the genotype 2a plasmid (strain JFH-1) was kindly provided by T. Pietschmann and R. Bartenschlager (University of Heidelberg, Germany). Plasmids encoding the vesicular stomatitis virus glycoprotein G and feline endogenous virus RD114 glycoprotein [70] were used for the production of VSVpp and RD114pp, respectively. In each experiment, pseudotyped particles produced in the absence of envelope proteins were used as controls. The mean luminescence activity of such particles represented less than 2% of the activity measured for HCVpp. In cholesterol depletion and Smase experiments, particles were produced in DMEM containing 2% lipoprotein-depleted serum (LPDS) [71]. At 40–48 h post-infection, cells were lysed and processed to measure the *Firefly *luciferase activities as indicated by the manufacturer (Promega). Luciferase activities were normalized for protein concentration in each cell lysate. In each figure, results are reported as the mean ± S.D. of three independent experiments.

### Detection of cell surface biotinylated proteins

Cells were biotinylated with 0.2 mg/mL EZ-link-Sulfo-NHS-LC-biotin (Pierce) in Hanks buffered saline solution (Invitrogen) for 30 minutes at 4°C. After 3 rinses with PBS 0.6% Bovine Serum Albumin (BSA, Euromedex), cells were lysed in lysis buffer (1% Brij97 in D-PBS with Ca and Mg or 1% Triton X-100 in D-PBS with 2 mM EDTA) containing protease inhibitors (Complete, Roche). Lysates were precleared for 2 h at 4°C with protein A-sepharose (Amersham Biosciences), then incubated for 2 h at 4°C with specific mAbs immobilized onto protein A-sepharose beads. After rinsing with the lysis buffer, complexes were eluted with non-reducing Laemmli buffer, resolved by SDS-PAGE and immunoblotted with peroxidase-conjugated Streptavidin (Vector).

### Statistical analyses

The Mann-Whitney's test, based on ranks, was used to compare the results to the reference. The reported p-values were asymptotic and two-sided. We considered a difference as significant for any p-value < 0.05. The tests were performed with the software SPSS 14.0.2.

### Flow cytometry analysis

Cells were rinsed with PBS 2% Bovine Serum Albumin (PBS/BSA) and incubated for 1 h at 4°C with anti-human CD81 (1.3.3.22), anti-murine CD81 (MT81, MT81w) or anti-human CD151 (TS151) mAbs. After rinsing with PBS/BSA, cells were stained with phycoerythrin (PE) labeled goat anti-mouse or anti-rat (BD Pharmingen) for 45 min at 4°C. After rinsing, cells were detached with PBS 2 mM EDTA and fixed with Formalin Solution (Sigma). Cells stained only with the secondary antibodies were used as negative control. Labelled cells were analyzed using a FACS Beckman EPICS-XL MCL.

## Authors' contributions

VRP, ML, DD, JD, CW and LC conceived and designed the experiments. VRP, ML, DD, JC, AP, JP, CW and LC performed the experiments. CL performed the statistical analyses. ER, JD, CW and LC contributed to reagents/materials/analysis tools. VRP, ML and LC wrote the paper.

## Authors' information

JD is an international scholar of the Howard Hughes Medical Institute.
